# Microbial Predominance and Antimicrobial Resistance in a Tertiary Hospital in Northwest China: A Six-Year Retrospective Study of Outpatients and Patients Visiting the Emergency Department

**DOI:** 10.1155/2020/8838447

**Published:** 2020-11-28

**Authors:** Caifeng Wang, Wen Li, Juanjuan Gao, Dancheng Zhang, Yali Li, Fang Li, Jine Lei

**Affiliations:** ^1^Department of Emergency, First Affiliated Hospital, Xi'an Jiaotong University, Xi'an, Shaanxi Province, China; ^2^Department of Clinical Laboratory, First Affiliated Hospital, Xi'an Jiaotong University, Xi'an, Shaanxi Province, China; ^3^Biological Specimen Management, First Affiliated Hospital, Xi'an Jiaotong University, Xi'an, Shaanxi Province, China; ^4^College of Laboratory Medicine, Chongqing Medical University, Chongqing, China

## Abstract

**Background:**

With the wide use of antibiotics, antimicrobial resistance becomes a serious issue. Timely understanding of microbial pathogen profiles and the change of antimicrobial resistance provide an important guidance for effective and optimized use of antibiotics in local healthcare systems. The aim was to investigate the characteristics of microbial species and their antimicrobial resistances in a tertiary hospital with an Emergency Department and outpatient clinics for a period of six years. *Methodology*. A retrospective study was conducted using the HIS database of a tertiary hospital between 2013 and 2018. Antimicrobial susceptibility was tested by automated systems and/or the Kirby–Bauer disc diffusion method. The data were analyzed using the WHONET 5.6 software. The Cochran-Armitage test was used to study the trends over the period of research.

**Results:**

In a total of 19,028 specimens submitted for microbial tests during the period from 49 units of the hospital, only the samples from the Emergency Department and Kidney Transplantation Clinic showed an annually significant increase (*P* < 0.001). More than 200 species with 46.4% gram-positive cocci and 45.3% gram-negative bacilli were identified in the 3,849 nonrepetitive isolates. The methicillin-resistant *S. aureus* and *S. epidermidis* rates were 25.1% and 74.6%, respectively. 60.9% *E. coli* and 33.5% *K. pneumonia* samples carried extended-spectrum-*β*-lactamase. All *Staphylococci* and *Enterococci* samples were not resistant to linezolid, vancomycin, and tigecycline. In addition, only 0.01% *E. coli*, 1.1% *K*. *pneumonia*, and 18.7% *P. aeruginosa* isolates showed resistance to carbapenems.

**Conclusions:**

Vancomycin, linezolid and tigecycline were the most effective antibiotics for outpatients with gram-positive infection. Carbapenems were the most effective antibiotics for gram-negative infection. There was no significant annual increase of common multidrug resistances.

## 1. Introduction

Increased usage of broad-spectrum antimicrobial treatments leads to microbial resistance to the treatments that were originally sensitive. The increased antimicrobial resistance (AMR) limits treatment options and is considered as a global challenge to public health with increased morbidity, mortality, and healthcare costs [1] To accurately estimate the challenge, the surveillance of AMR profiling at local, regional, and national levels has been employed to understand the global trends on the type of predominant pathogens and their respective resistance profiling [2]. In China, the surveillance of AMR is established at the hospital, provincial, and national levels. For example, CHINET is one of well-established national surveillance networks for AMR [3–6]. This network recently reported that the frequency of gram-negative bacilli among clinical isolates was over twice higher than that of gram-positive cocci [3, 4]. In addition, it is critical to recognize that China is a large country with variety of microbial occurrences and AMR profiling. For example, Xiao et al. reported that the percentage of MRSA (methicillin-resistant Staphylococcus aureus) in North Central and South Central China is higher than other regions [7]. The geographic variety is likely associated with regional differences in socioeconomic development [7]. Therefore, the microbial predominance and AMR profiling in a specific region and province is not always consistent with the trend at a national level. Indeed, the surveillance of AMR at the hospital and provincial levels is a valuable complement to the national surveillance. Furthermore, the updated AMR profiling at the hospital and regional levels is particularly important for physicians to determine the adequate empiric antimicrobial therapy in clinical practice. Therefore, the objectives of this study were to analyze the data from a six-year surveillance of AMR at an university affiliated tertiary hospital and to obtain the characteristics of microbial species and their antimicrobial resistances for both surveillance and clinical practice perspectives.

The routine surveillance was mainly focused on hospital-acquired infections, and most specimens were collected from inpatients with severe infections [8], while community-acquired infections from outpatient service were always underreported [2, 8]. Therefore, in our study, we tried to bridge the gap and studied the microbial predominance and AMR profiling in outpatient clinics and emergency department in a specific region of Northwestern China. Our results were also compared with the findings from the CHINET.

## 2. Materials and Methods

### 2.1. Data Source

This study was performed at the First Affiliated Hospital of Xi'an Jiaotong University, which is one of the largest hospitals in northwest China. It provides medical and surgical care to the residents of Shaanxi Province with a total population 37.33 million. The institutional review board at the First Affiliated Hospital approved this study (No: XJTU1AF2017LSK-83) and written informed consent was not required because the laboratory tests for microbiology are part of standard care and the patient records were excluded prior to this analysis. Archived laboratory data between 2013 and 2018 were retrieved from the HIS database of the hospital for analysis. A total of 19,028 specimens were collected from the emergency department and 49 outpatient clinics. A full list of these clinics is provided in Table 1. The specimens included urine, blood, prostatic fluid, sputum, pleural effusion and ascites, stool, dialysate, pus, secretion, drainage, cerebrospinal fluid, and bronchoalveolar lavage fluid.

### 2.2. Isolate Identification

The clinical specimens were processed according to the recommended microbiological procedures as previously described [9–12]. Species were identified through colony morphology, conventional biochemical reactions and/or the use of an automated system (bioMerieux, Marcy l'Etoile, France).

### 2.3. Antibiotic Sensitivity

Mueller-Hinton agar (MH agar) and MH agar with 5% sheep blood *Haemophilus* test medium (HTM) was purchased from bioMerieux, Marcy l'Etoile, France. The following ATCC strains were used as references: *Escherichia coli* (ATCC 25922), *Pseudomonas aeruginosa* (ATCC 27853), *Staphylococcus aureus* (ATCC 25923), and *Enterococcus faecallis* (ATCC 29212). An automated system (bioMerieux, Marcy l'Etoile, France) and/or the Kirby–Bauer Disc Diffusion Method were used to test for antimicrobial susceptibility according to the guidelines of the Clinical Laboratory Standards Institute. The sensitivity breakpoint of cefoperazone/sulbactam referred to that of cefoperazone.

The following antibiotics were tested against available isolates: piperacillin, oxacillin, ampicillin, ampicillin/sulbactam, piperacillin/tazobactam, cefoperazone/sulbactam, cefazolin, cefuroxime, ceftriaxone, cefepime, ciprofloxacin, levofloxacin, moxifloxacin, amikacin, gentamicin, high level gentamicin, tobramycin, aztreonam, erythromycin, clindamycin, vancomycin, linezolid, tetracycline, trimethoprim/sulfamethoxazole, and tigecycline.

### 2.4. Statistical Analyses

Data from the isolates and the susceptibility testing were analyzed using the WHONET 5.6 software provided by the World Health Organization. The Cochran-Armitage test was used to study the trends of the specimen numbers submitted from different outpatient clinics, different specimen types, and the antibiotic resistance percentages over time. This statistical analysis was performed using R 3.6.1 (R Foundation, Vienna, Austria) and a two-sided *P*-value 0.05 was considered as statistically significant.

## 3. Results

### 3.1. Specimens' Information

From 2013 to 2018, a total of 19,028 specimens were submitted to the clinical microbiology laboratory from 49 outpatient units. The total number of specimens increased from 2,303 in 2013 to 5,378 in 2018. As observed in Table 1, the number of specimens submitted from each department showed an increase over time. The top three units with the largest number of submissions were Emergency (30.7%), Urology (18.9%), and Nephrology (16.0%). However, only the Emergency and Kidney Transplantation Clinic showed significant increases in the percentage of specimens submitted annually (*P* < 0.001). Notably, the data from the Breast Surgery Clinic were incomplete and therefore were not included in the analysis. There were no significant differences for the percentages of specimen submission from the Peritoneal Dialysis Clinic. In contrast, all other departments showed a significant decrease in the annual percentage of specimen submission (Table 1).

### 3.2. The Different Types of Specimens Submitted for the Laboratory Tests of Microbiology

As shown in Table 2, the number of submitted specimens increased annually among different types of specimens, which was consistent with the increase of total number of specimen submission each year in Table 1. Sputum, whole blood, and pus showed a significant increase of specimen submission annually (*P* < 0.001). In contrast, urine, prostatic fluid, and other types of specimens showed a significant decrease of specimen submission annually (*P* < 0.001). The submissions of dialysate, peritoneal drainage, and pleural effusion and ascites had no significant changes over the period (*P* > 0.05).

### 3.3. The Microbial Growth between Different Types of Specimens

In a total of 19,028 clinical specimens, 4,719 (24.8%) were tested positive for bacterial and fungal growth. The specimen types with high positive rates were peritoneal drainage (67% (219/327)), dialysate (52.7% (267/507)), pus (49.3% (250/507)), and pleural effusion and ascites (31.9% (60/188)), followed by urine (28.9% (1,843/6, 383)), prostatic fluid (28.1% (528/1,881)), whole blood (12.9% (405/3,139)), and sputum (9.7% (323/3,314)).

### 3.4. Common Microbial Isolates and Species

In a total of 4,719 isolates of bacteria and fungi identified, after excluding duplicate isolates obtained from the same patient (each patient was sampled only once), there were 3,849 nonrepetitive isolates. These isolates belong to 211 different species, including 1,786 Gram-negative isolates (44.6%), 1,744 Gram-positive isolates (45.3%), 150 fungus isolates (3.9%), and 169 isolates containing other species (4.4%). The most frequently identified Gram-negative species were *Escherichia coli, Klebsiella pneumonia* and *Pseudomonas aeruginosa, Enterobacter cloacae, Acinetobacter baumannii, and Proteus mirabilis.* The most frequently identified Gram-positive species were *Staphylococcus epidermidis, Staphylococcus aureus, Enterococcus faecalis, Staphylococcus haemolyticus, Streptococcus mitis,* and *Enterococcus faecium*.  Table 3 shows the number and percentage (%) of bacterial isolates from the outpatient specimens annually between 2013 and 2018. The percentage of *S. aureus* (*P*=0.002) and *E. cloacae* (*P*=0.033) demonstrated a significant increase annually, while *S. epidermidis* (*P* < 0.001) and *S. haemolyticus* (*P* < 0.001) showed a decrease. *Candida albicans* (78 isolates) and *Aspergillus fumigatus* (24 isolates) were the major fungi isolated, followed by *Aspergillus flavus* (9 isolates), Genus of *Mucor* and *Fusarium* (9 isolates), and other *Candida* species (30 isolates). Other rare bacterial isolates were *Corynebacterium* (109 isolates), *Lactobacillus* (36 isolates), anaerobic bacteria (13 isolates) as well as *Brucella, Eikenella, Actinomyces, Nocardia,* and *Nontuberculous Mycobacterium.* Among *Corynebacterium*, *Kroppenstedt Corynebacterium* (23 isolates), and *Ribbone Corynebacterium* (10 isolates) were predominant.

### 3.5. The Antimicrobial Resistance Profile of the Common Pathogens

#### 3.5.1. *Staphylococcus* spp

Our study indicated that all of *S. aureus* isolates in our community were resistant to penicillin (Table 4). However, many *S. aureus* strains, while resistant to penicillin, remained susceptible to penicillinase-stable penicillin, such as oxacillin. Strains resistant to oxacillin and methicillin were historically termed methicillin-resistant *S. aureus* (MRSA) [13]. Our study found that the percentage of MRSA isolates was 25.1% during the study period (Table 4) without significant changes annually (Table 5). In contrast, the CHINET reported that the prevalence of MRSA decreased from 69.0% in 2005 to 35.2% in 2017 [3]. The difference between our study and the CHINET is likely due to the lower prevalence of MRSA in community-acquired infections over the period in our study, suggesting the infiltration of MRSA isolates from hospitals to the community is very limited.

As shown in Table 4, less than 30% of *S. aureus* isolates were resistant to trimethoprim/sulfamethoxazole (29.5%), gentamicin (23.3%), ciprofloxacin (17.6%), levofloxacin (17.6%), and moxifloxacin (6.6%), indicating a minor resistance to fluoroquinolones and aminoglycosides. In comparison, more than 30% of *S. aureus* isolates were resistant to erythromycin (70.5%), clindamycin (41.4%) and tetracycline (41.4%). Like *S. aureus*, almost all of *S. epidermidis* isolates (96.6%) in our community-acquired infections were resistant to penicillin (Table 4). Strains of *S. epidermidis* that were resistant to oxacillin and methicillin were historically termed methicillin-resistant *S. epidermidis* (MRSE). Our study showed that the percentage of MRSE isolates was 74.6% during the study period (Table 4) without significant changes annually (Table 5). Less than 30% of *S. epidermidis* isolates were resistant to clindamycin (22.0%), tetracycline (20.1%), gentamicin (8.6%) and moxifloxacin (7.5%), whereas more than 30% of *S. epidermidis* isolates were resistant to erythromycin (82.1%), compound sulfamethoxazole (64.6%), levofloxacin (40.7%), and ciprofloxacin (32.1%). These findings indicate a moderate resistance of *S. epidermidis* to fluoroquinolones and a minor resistance to aminoglycosides. Significantly, all of *S. epidermidis* and *S. aureus* isolates collected from our study were sensitive to vancomycin, linezolid, and tigecycline.

#### 3.5.2. *Enterococcus* spp

Among *Enterococcus* species, two major species (*Enterococcus faecalis* and *Enterococcus faecium*), were particularly human-specific pathogens. As indicated in Table 4, although the number of isolates of *E. faecalis* was significantly higher than that from *E. faecium* (193 isolates vs. 94)*, E. faecalis* isolates were less resistant to most antibiotics compared to *E. faecium* isolates, including ampicillin (0.0% vs. 94.7%), erythromycin (57.0% vs. 90.4%), ciprofloxacin (23.8% vs. 96.8%), penicillin (0.0% vs. 94.7%), and levofloxacin (22.8% vs. 94.7%); the only exception was tetracycline (66.8% vs. 45.7%). Furthermore, all of *E. faecalis* and *E. faecium* isolates collected from our study were sensitive to linezolid, tigecycline, and vancomycin.

#### 3.5.3. Enterobacteriaceae

Enterobacteriaceae, including *E. coli* and *K. pneumonia*, produce extended-spectrum *β*-lactamases (ESBLs). ESBLs are a group of *β*-lactamases, which share the ability to hydrolyze *β*-lactam antibiotics, such as cephalosporins [14]. By using ceftriaxione as a substrate, our study suggested that 60.9% of *E. coli* isolates and 33.5% of *K. pneumonia* carried ESBLs (Table 6). Consistent with the ESBLs results, a similar trend was observed for the percentage of *E. coli* and *K. pneumonia* isolates resistant to other cephalosporins: cefepime (33.0% vs. 5.9%), cefuroxime (80.7% vs. 23.5%), ceftazidime (54.5% vs. 17.6%), and cefazolin (79.5% vs. 17.6%) with the exception of cefotetan (5.7% vs. 0.0%) (Table 6). Less than 30% of *E. coli* isolates were resistant to tobramycin (21.6%), cefoperazone/sulbactam (8.4%), piperacillin/tazobactam (8.0%), and amikacin (2.3%). More than 30% of *E. coli* isolates were resistant to ampicillin (93.2%), piperacillin (86.4%), ciprofloxacin (80.7%), levofloxacin (76.1%), ampicillin/shubatan (70.5%), trimethoprim/sulfamethoxazole (62.5%), aztreonam (60.2%), and gentamicin (39.8%), indicating a high resistance to penicillins and fluoroquinolones. All of *E. coli* isolates were sensitive to imipenem and meropenem. As shown in Table 6, less than 30% of *K. pneumonia* isolates were resistant to compound sulfamethoxazole (29.4%), ampicillin/shubatan (23.5%), piperacillin (17.6%), ciprofloxacin (11.8%), levofloxacin (11.8%), aztreonam (5.9%), gentamicin (5.9%), imipenem (1.1%), and meropenem (1.1%), indicating a minor resistance to these common antibiotics, such as penicillins and fluoroquinolones. All of the *K. pneumonia* isolates were sensitive to amikacin, piperacillin/tazobactam, cefoperazone/sulbactam, cefotetan, and tobramycin.

#### 3.5.4. Nonfermentative Bacteria

All of *P. aeruginosa* isolates were sensitive to fluoroquinolones, including ciprofloxacin and levofloxacin. The *P. aeruginosa-*resistant isolates to other antibiotics were all less than 30% including aztreonam (22.4%), metopenem (18.7%), imipenem (18.7%), ceftazidime (15.0%), piperacillin (14.9%), cefoperazone/sulbactam (10.3%), amikacin (7.5%), piperacillin/tazobactam (7.5%), gentamicin (7.5%), cefepime (7.5%), and tobramycin (7.5%), suggesting a minor resistance to these common antibiotics.

## 4. Discussion

In this study, we reported the microbial predominance and AMR profiling in a tertiary hospital using retrospective data from outpatient clinics and emergency department in a specific region of Northwestern China over six years. We will discuss our findings in the following five aspects.

### 4.1. The Medical Value to Study the Distribution of Specimen Submitted between Different Outpatient Clinics and Emergency Department

There are limited publications that compared the number and percentage of specimens submitted to clinical microbiology laboratories between different outpatient clinics. Different from other countries, patients in China can directly visit healthcare services without referral requirements from primary care doctors. Our study showed a trend of increasing number of specimen submissions in each clinic (Table 1), likely associated with the increased population in the communities. The departments of Emergency, Urology, and Nephrology were among the top three clinics that submitted specimens for microbiological studies. Urinary tract infection has been considered the major reason for outpatients to visit the Urology and Nephrology departments [15] and is also one of the major concerns for the Emergency visits [16]. More importantly, our study revealed that only two departments, the Emergency Department and Kidney Transplantation Clinic, demonstrated a significant increase of specimen submitted annually (*P* < 0.001). This indicates that these two departments, particularly the Emergency Department [17], are in the frontline of infection control. The increased importance of the Emergency Department is likely associated with enhanced medical functions of the emergency service. There is a trend for the Emergency Department to handle more previously inpatient cares. The increased importance of the Kidney Transplantation Department indicates that Kidney Transplantation is becoming a mainstream medical service and repeated infection after kidney transplantation is an urgent medical issue in outpatient clinics [18].

### 4.2. Relatively Lower Ratio of Gram-Negative Isolates in Our Community-Acquired Infections

Among 3,849 nonrepetitive isolates, the ratio of Gram-negative to Gram-positive was nearly 1:1 (45.3% vs. 46.4%) in our study while the ratio reported by the CHINET was roughly 2:1 with approximately 70% of Gram-negative bacteria [3, 4]. This difference is probably because that the CHINET data were significantly derived from inpatient service and our data were from a mixture of outpatient and inpatient services. Studies from US hospitals reported that Gram-negative bacteria were more common in cases with ventilator-associated pneumonia and urinary tract infections [19] and Gram-negative bacteria were the dominant type of infection at the intensive care units [20]. These results suggest that there is a likely association between the higher ratio of Gram-negative infections and the inpatients experiencing invasive medical devices and surgical procedures. Indeed, the Gram-negative bacteria contain highly efficient mechanisms of antibiotic drug resistance [21]. By comparing Tables 4 and 6 in this study, Gram-negative bacteria, particularly *E. coli*, appeared more resistant to the antibiotics and required more complicated therapeutic regimens. Overall, relatively lower ratio of Gram-negative isolates was likely a general feature in outpatient service.

### 4.3. Vancomycin, Linezolid, and Tigecycline Were the Most Effective Antibiotics for Patients with Gram-Positive Infection in Our Community

Vancomycin, linezolid, and tigecycline have been used to treat multidrug resistant bacteria in the community [22]. In our study, *Staphylococci* and *Enterococci* (Table 4) were 100% sensitive to vancomycin and linezolid, which was in general consistent with the findings from the CHINET. For example, the CHINET surveillance in 2018 showed that *Staphylococci* isolated from blood, urine, the lower respiratory tract, and cerebrospinal fluid were 100% sensitive to vancomycin and linezolid except less than 1% of *Staphylococci* isolated from blood was resistant to linezolid [4]. Moreover, less than 5% of *E. faecium* and less than 1% of *E. faecalis* were vancomycin-resistant in the CHINET surveillance from 2005 to 2017 [3]. These findings indicate that vancomycin and linezolid remain the most effective treatment for Gram-positive infection for both hospital and community acquired infections.

In our study, *Staphylococci* and *Enterococci* (Table 4) were also 100% sensitive to tigecycline, which was an expanded broad-spectrum intravenous glycylcycline antibiotic. This finding suggests that tigecycline is a primary antibiotic treatment for multidrug-resistant bacterial infection in our communities.

### 4.4. Carbapenems Were the Most Effective Treatment for Patients with Gram-Negative Infections in Our Communities

Table 6 shows higher resistance rates of Gram-negative pathogens, particularly *E. coli*, to the cephalosporins and quinolones in this study. To overcome the clinical challenge, carbapenems such as imipenem and meropenem have been used for these multidrug-resistant bacterial infections [23]. Table 6 also shows that the percentages of *E. coli*, *K. pneumonia* and *P. aeruginosa* isolates resistant to imipenem were 0.0%, 1.1%, and 18.7%, respectively. Moreover, the annual rate of resistance of *E. coli* and *K. pneumonia* to carbapenems showed no significant increase as shown in Table 5. These findings support that carbapenems should be a primary antibiotic for multidrug-resistance bacterial infections in our communities.

### 4.5. There Was No Significant Increase in Multidrug-Resistant Bacteria Observed in Our Communities

Tables 4 and 6 show higher resistance rates between certain bacteria and antibiotics, such as methicillin-resistant Gram-positive pathogens (MRSA and MRSE) and extended spectrum *β* lactamases (ESBLs)-resistant Gram-negative pathogens. By using ceftriaxone and oxacillin, Table 5 indicates an annual change of the resistance rate of multidrug-resistant bacteria in recent years. A significant increase of resistance rate would require immediate intervention to investigate the reason. The results in Table 5 suggest that there is no significant increase of multidrug-resistant bacteria, indicating a reasonable prescription of antibiotics in our outpatient and emergency services and successful physician education on the prevention of antibiotic resistance. The main limitation in this study was that this was a single-center retrospective observational study. It should be cautious to translate our results to other hospitals. However, the quality of clinical sampling procedures and techniques were in general better controlled in a single center than multiple centers. First, they also allowed us to obtain a representative specimen in many clinical departments throughout the hospital. When we repeated the sample collection and analysis annually, we could observe trends over a period of time. In addition, a single-center study, when appropriately performed, is a useful and inexpensive surveilling tool to reflect the regional situation of prevailing microorganisms and their resistance to antimicrobials. Second, outpatient surveillance is an important tool to study community-acquired infections. It should be emphasized that not all outpatient and inpatients visiting the Emergency Department with infections are contracted from the communities. This is the reason we only used the term of “community settings” in this study since the patients seeking medical care in our outpatient and emergency services come directly from the community.

## Figures and Tables

**Table 1:** Annual number and percentage (%) of the specimens submitted from outpatient clinics and emergency department for the laboratory tests from 2013 to 2018.

Department	2013		2014		2015		2016		2017		2018		*P* value	Trend
	*N*	%	*N*	%	*N*	%	*N*	%	*N*	%	*N*	%		
Emergency	469	20.4	490	19.7	517	21.0	1017	32.5	1046	32.0	2.306	42.9	<0.001	↑
Urology	400	17.4	710	28.5	497	20.1	426	13.6	673	20.6	891	16.6	<0.001	↓
Nephrology	559	24.3	427	17.2	491	19.9	511	16.3	466	14.3	581	10.8	<0.001	↓
Respiratory	272	11.8	211	8.5	381	15.4	491	15.7	300	9.2	475	8.8	0.002	↓
Convenient^*∗*^	120	5.2	129	5.2	134	5.4	126	4.0	115	3.5	160	3.0	<0.001	↓
Rheumatology	150	6.5	71	2.9	56	2.3	68	2.2	85	2.6	124	2.3	<0.001	↓
Peritoneal dialysis	45	2.0	91	3.7	82	3.3	64	2.0	69	2.1	115	2.1	0.083	No change
Kidney transplant	33	1.4	52	2.1	25	1.0	36	1.2	69	2.1	143	2.7	0.0043	↑
Gynecology	32	1.4	74	3.0	80	3.2	68	2.2	50	1.5	53	1.0	<0.001	↓
Breast surgery	*∗∗*	*∗∗*	*∗∗*	*∗∗*	*∗∗*	*∗∗*	62	2.0	103	3.2	140	2.6	*∗∗*	*∗∗*
Other^*∗∗∗*^	223	9.7	232	9.3	204	8.3	258	8.3	290	8.9	390	7.3	0.012	↓
Total	2.303	100.0	2.487	100.0	2.467	100.0	3.127	100.0	3.266	100.0	5.378	100.0		

^*∗*^Convenient clinic helps to meet simple requests of the patients with frequent visits, such as to fill in the prescription and/or to obtain the regular laboratory tests. ^*∗∗*^Data not available. ^*∗∗∗*^Other outpatient clinics include dermatology clinic, blood purification clinic, infectious disease clinic, hematology clinic, oncology clinic, pediatric clinic, Chinese medicine clinic, digestive medicine clinic, otolaryngology clinic, general surgery clinic, orthopedic surgery clinic, endocrinology clinic, ophthalmology clinic, thoracic surgery clinic, hepatobiliary surgery clinic, neurology clinic, obstetrics clinic, cardiovascular disease clinic, cardiac and vascular clinic, neurosurgery clinic, geriatric surgery clinic, oncology surgery clinic, Chinese medicine clinic (Xingshan campus), neonatal clinic, chest clinic, burn plastic surgery clinic, reproductive medicine clinic, cardiovascular surgery clinic, biotherapy consultation clinic, stomatology clinic, general medicine clinic, tumor radiotherapy clinic, structural heart disease clinic, famous senior Chinese medicine clinic, peripheral vascular clinic, rehabilitation clinic (Xingshan campus), Lugang obstetrics and gynecology clinic, pain clinic, and vascular surgery clinic.

**Table 2:** Annual number and percentage (%) of the types of specimens submitted for the laboratory tests from outpatients and patients visiting emergency department between 2013 and 2018.

Specimen type	2013		2014		2015		2016		2017		2018		*P* value	Trend
	*N*	%	*N*	%	*N*	%	*N*	%	*N*	%	*N*	%		
Urine	919	39.9	778	31.3	805	32.6	970	31.0	1.178	36.1	1.733	32.2	0.025	↓
Sputum	395	17.2	262	10.5	393	15.9	672	21.5	515	15.8	1.077	20.0	<0.001	↑
Whole blood	231	10.0	284	11.4	276	11.2	511	16.3	636	19.5	1.201	22.3	<0.001	↑
Prostatic fluid	248	10.8	491	19.7	321	13.0	220	7.0	254	7.8	348	6.5	<0.001	↓
Pus^*∗*^	29	1.3	33	1.3	43	1.7	81	2.6	142	4.3	179	3.3	<0.001	↑
Dialysate	29	1.3	85	3.4	76	3.1	74	2.4	96	2.9	147	2.7	0.235	No change
Peritoneal drainage	9	0.4	52	2.1	45	1.8	82	2.6	91	2.8	48	0.9	0.709	No change
Pleural effusion and ascites	8	0.3	25	1.0	17	0.7	52	1.7	31	0.9	55	1.0	0.117	No change
Others^*∗∗*^	435	18.9	477	19.2	491	19.9	465	14.9	323	9.9	590	11.0	<0.001	↓
Total	2.303	100.0	2.487	100.0	2.467	100.0	3.127	100.0	3.266	100.0	5.378	100.0		

^*∗*^Pus was collected from skin and mammary gland. ^*∗∗*^Others mainly include cerebrospinal fluid, joint fluid, feces, reproductive tract secretions and bone marrow.

**Table 3:** Annual number and percentage (%) of bacterial isolates from the specimens between 2013 and 2018 from outpatients and patients visiting emergency department.

Bacterial species	2013		2014		2015		2016		2017		2018		*P* value	Trend
	*N*	%	*N*	%	*N*	%	*N*	%	*N*	%	*N*	%		
Gram-negative														
*Escherichia coli*	134	70.2	149	66.8	128	61.0	138	53.5	218	60.2	268	56.9	0.471	No change
*Klebsiella pneumonia*	21	11.0	24	10.8	14	6.7	29	11.2	38	10.5	56	11.9	0.400	No change
*Pseudomonas aeruginosa*	9	4.7	19	8.5	11	5.2	18	7.0	18	5.0	32	6.8	0.760	No change
*Enterobacter cloacae*	4	2.1	3	1.3	1	0.5	9	3.5	12	3.3	22	4.7	0.033	↑
*Acinetobacter baumannii*	6	3.1	5	2.2	7	3.3	7	2.7	9	2.5	14	3.0	0.870	No change
*Proteus mirabilis*	3	1.6	6	2.7	5	2.4	9	3.5	11	3.0	7	1.5	0.985	No change
Others^*∗*^	14	7.3	17	7.6	44	21.0	48	18.6	56	15.5	72	15.3		
Total	191	100.0	223	100.0	210	100.0	258	100.0	362	100.0	471	100.0		

Gram-positive														
*Staphylococcus epidermidis*	37	18.1	81	28.1	37	20.4	29	12.7	45	18.0	39	11.3	<0.001	↓
*Staphylococcus aureus*	12	5.9	20	6.9	20	11.0	45	19.7	50	20.0	80	23.3	0.002	↑
*Enterococcus faecalis*	21	10.3	33	11.5	20	11.0	30	13.2	33	13.2	56	16.3	0.984	No change
*Staphylococcus haemolyticus*	34	16.7	39	13.5	22	12.2	12	5.3	11	4.4	20	5.8	<0.001	↓
*Streptococcus mitis*	5	2.5	16	5.6	16	8.8	17	7.5	20	8.0	28	8.1	0.556	No change
*Enterococcus faecium*	9	4.4	7	2.4	10	5.5	17	7.5	19	7.6	32	9.3	0.163	No change
Others^*∗∗*^	86	42.2	92	31.9	56	30.9	78	34.2	72	28.8	89	25.9		
Total	204	100.0	288	100.0	181	100.0	228	100.0	250	100.0	344	100.0		

^*∗*^Other gram-negative bacteria mainly include *Enterobacteria*, *Pseudomonas*, *Proteus*, *Citrobacter*, *Haemophilus*, *Neisseria*, *Bacteroides*, *Aeromonas*, and *Morgan* bacteria. ^*∗∗*^Other positive bacteria mainly include coagulase-negative *Staphylococci, Streptococci, Enterococci, Micrococcus, Cooka, Lactobacillus, Corynebacterium, Actinomycete, Nocardia*, and *Clostridia*.

**Table 4:** Antimicrobial resistance percentage (%) of top four Gram-positive cocci among common antimicrobial agents between 2013 and 2018 from outpatients and patients visiting emergency department.

Antimicrobial agent	*Staphylococcus aureus* (%) (*n* = 227)^*∗*^	*Staphylococcus epidermidis* (%) (*n* = 268)^*∗*^	*Enterococcus faecalis* (%) (*n* = 193)^*∗*^	*Enterococcus faecium* (%) (*n* = 94)^*∗*^
Penicillins				
Penicillin G	100.0	96.6	0.0	94.7
Oxacillin	25.1	74.6	^*∗∗*^	^*∗∗*^
Ampicillin	^*∗∗*^	^*∗∗*^	0.0	94.7

Fluoroquinolones				
Ciprofloxacin	17.6	32.1	23.8	96.8
Levofloxacin	17.6	40.7	22.8	94.7
Moxifloxacin	6.6	7.5	^*∗∗*^	^*∗∗*^

Aminoglycosides				
Gentamicin	23.3	8.6	^*∗∗*^	^*∗∗*^
Gentamicin HLAR	^*∗∗*^	^*∗∗*^	0.0	0.0

Others				
Erythromycin	70.5	82.1	57	90.4
Clindamycin	41.4	22	^*∗∗*^	^*∗∗*^
Vancomycin	0.0	0.0	0.0	0.0
Linezolid	0.0	0.0	0.0	0.0
Tetracycline	41.4	20.1	66.8	45.7
Trimethoprim/sulfamethoxazole	29.5	64.6	0.0	0.0
Tigecycline	0.0	0.0	0.0	0.0

^*∗*^The number of non-repetitive isolates for each species. ^*∗∗*^The result is not available.

**Table 5:** Annual multidrug resistance percentage (*R*%) of bacterial isolates from the specimens between 2013 and 2018 from outpatients and patients visiting emergency department.

Bacterial species	Years	2013	2014	2015	2016	2017	2018	*P* value	Trend
	Multidrug resistance targets	*R*%	*R*%	*R*%	*R*%	*R*%	*R*%		
*Escherichia coli*	ESBLs^*∗*^	62.6	59.6	57.4	59.3	56.8	59.4	0.586	No change
	CRE^*∗∗*^	0.0	0.0	0.0	0.0	0.0	0.0	—	No change
*Klebsiella pneumoniae*	*ESBLs* ^*∗*^	29.2	40.5	20.0	30.0	39.3	32.5	0.935	No change
	CRE^*∗∗*^	0.0	0.0	0.0	0.0	1.8	1.3	0.443	No change
*Staphylococcus aureus*	MRSA^*#*^	53.8	27.6	25.9	25	25	19.2	0.191	No change
*Staphylococcus epidermidis*	MRSE^*##*^	88.1	83.3	78.7	94.1	72.7	75.6	0.213	No change

^*∗*^ESBLs (extended spectrum *β* lactamases): ESBLs multidrug resistance was evaluated by using ceftriaxone. ^*∗∗*^CRE (carbapenem-resistant enterobacteriaceae): CRE multidrug resistance was evaluated by using imipenem and meropenem. ^*#*^MRSA (methicillin-resistant S. aureus): MRSA multidrug resistance was evaluated by using oxacillin. ^*##*^MRSE (methicillin-resistant S. epidermidis): MRSE multidrug resistance was evaluated by using oxacillin.

**Table 6:** Antimicrobial resistance percentage (%) of top three Gram-negative bacilli among common antimicrobial agents between 2013 and 2018 from outpatients and patients visiting emergency department.

Antimicrobial agent	*Escherichia coli* (%) (*n* = 1035)^*∗*^	*Klebsiella pneumoniae* (%) (*n* = 182)^*∗*^	*Pseudomonas aeruginosa* (%) (*n* = 107)^*∗*^
Penicillins			
Piperacillin	86.4	17.6	14.9
Ampicillin	93.2	_^*∗∗*^	−^*∗∗*^
Cephems			
Cefazolin	79.5	17.6	−^*∗∗*^
Cefuroxime	80.7	23.5	−^*∗∗*^
Ceftriaxone	60.9	33.5	−^*∗∗*^
Ceftazidime	54.5	17.6	15.0
Cefepime	33.0	5.9	7.5
Cefotetan	5.7	0.0	_^*∗∗*^
*β*-lactam combination agents			
Ampicillin/Shubatan	70.5	23.5	_^*∗∗*^
Piperacillin/Tazobactam	8.0	0.0	7.5
Cefoperazone/sulbactam	8.4	0.0	10.3
Fluoroquinolones			
Ciprofloxacin	80.7	11.8	0.0
Levofloxacin	76.1	11.8	0.0
Aminoglycosides			
Amikacin	2.3	0.0	7.5
Gentamicin	39.8	5.9	7.5
Tobramycin	21.6	0.0	7.5
Others			
Aztreonam	60.2	5.9	22.4
Imipenem	0.0	1.1	18.7
Meropenem	0.0	1.1	18.7
Trimethoprim/sulfamethoxazole	62.5	29.4	_^*∗∗*^

^*∗*^The number of non-repetitive isolates for each species. ^*∗∗*^The result is not available.

## Data Availability

All the data are available upon request.
